# The effects of a fermented rapeseed meal or/and soybean meal additive on the blood lipid profile and immune parameters of piglets and on minerals in their blood and bone

**DOI:** 10.1371/journal.pone.0253744

**Published:** 2021-06-24

**Authors:** Anna Czech, Eugeniusz R. Grela, Bożena Nowakowicz-Dębek, Łukasz Wlazło

**Affiliations:** 1 Department of Biochemistry and Toxicology, Faculty of Animal Sciences and Bioeconomy, University of Life Sciences in Lublin, Lublin, Poland; 2 Institute of Animal Nutrition and Bromatology, Faculty of Animal Sciences and Bioeconomy, University of Life Sciences in Lublin, Lublin, Poland; 3 Department of Animal Hygiene and Environmental Hazards, Faculty of Animal Sciences and Bioeconomy, University of Life Sciences in Lublin, Lublin, Poland; West Virginia University, UNITED STATES

## Abstract

The aim of the study was to assess the effect of the inclusion of dried fermented soybean and/or rapeseed meal in piglet feed on immune parameters, blood lipid parameters, and mineral content in the blood and metacarpal bones. The study was conducted on 150 28-day-old piglets divided into 5 groups. Piglets in the control group (C) received a standard diet with soybean meal. Animals in group FR (group receiving a diet with 8% FRSM) received a diet in which a portion of the soybean meal was replaced with 8% dried fermented rapeseed meal (FRSM). Animals in group FR/FS received a diet in which a portion of the soybean meal was replaced with 6% FRSM and 2% fermented dried soybean meal (FSBM). The piglets in group FS/FR received a diet with 6% FSBM and 2% FRSM. Group FS received a diet in which a portion of the soybean meal was replaced with an 8% share of FSBM. The inclusion of 8% or 6% fermented rapeseed meal (group FR or FR/FS) in the diet of piglets had a beneficial effect on their immune status, as evidenced by the increase in plasma levels of IgG and IgA and the decrease in IL-6 relative to the control group. It also significantly increased the concentrations of minerals, i.e. P, Ca and Zn, in the blood plasma and metacarpal bones of piglets and improved the availability of iron, a key bioelement involved in haemoglobin. The use of 8% or 6% fermented soybean meal in the diet (groups FS and FS/FR) of piglets had a positive effect on blood lipid parameters, reducing CHOL and LDL-cholesterol in the plasma. In conclusion, the fermentation process enables better utilization of rapeseed or soybean meal by pigs. Dried fermented rapeseed meal could partially replace protein components from GMO (genetically modified ogranism) crops (soybean meal) used in diets for pigs.

## Introduction

Rapeseed and soybean meal are important protein sources that can be used in feeding livestock. Their wider use, especially for monogastric animals, is impeded by the presence of numerous anti-nutritional factors, such as glucosinolates, protease inhibitors, tannins, phytic acid and, in the case of rapeseed meal, relatively high crude fibre content [1]. It is primarily young individuals, whose digestive and defence functions are not fully developed, that are susceptible to the anti-nutritional effects of these feed components. Recent research shows that fermentation can be one of the technological methods for improving the nutritional value of soybean and rapeseed meals and their suitability as feed [2]. The nutritional benefits of this type of feed may result in better livestock production [2, 3]. Research by Canibe and Jensen [4] shows that fermented feed components have health-promoting properties, as a source of probiotic microbes, digestive enzymes, and antioxidant compounds. The presence of these compounds has a beneficial effect on the intestinal microbiota, which in turn improves immune status [5, 6]. Fermented feed influences not only the humoral response (Ig content, lysozyme activity, and cytokine production), but also cellular immunity [1]. The decrease in the ratio of heterophils to lymphocytes observed in chickens fed fermented products suggests that they may alleviate oxidative stress, which may lead to suppression of the immune response [7]. In addition, the population of lactic acid bacteria introduced with fermented products is responsible for the production of short-chain fatty acids, creating a competitive environment protecting against infection and pathogenic bacteria such as *Salmonella* and coliforms [8].

Fermented feedstuffs, due to the presence of lactic acid, other short-chain organic acids, and probiotic bacteria, in addition to degradation of phytate complexes, improve the absorption of amino acids and minerals, primarily phosphorus and calcium, which can modify metabolic processes in the body. This is reflected in the haematological and biochemical profile of the blood [3, 9]. This relationship is confirmed by research by Fazhi et al. [10], who indicate that the activity of microbial phytase, which reduces chelate complexes of phytic acid with minerals, is higher in fermented components. The inclusion of a fermented component in the diet of piglets may also improve synthesis of amino acids, e.g. those essential to the formation of collagen in bones–glutamine and proline [11]. Moreover, it can increase the absorption of haematopoietic elements (iron and copper), which affect erythropoiesis and also influence the metabolism of lipid components [12].

In view of the above, we conducted research to verify the hypothesis that the inclusion of dried fermented protein feedstuffs in the diet of piglets can have a beneficial effect on their immunity and blood lipid parameters, and also improve the bioavailability of minerals.

The aim of the study was to assess the effect of dried fermented rapeseed and/or soybean meal in the diet of piglets on immune parameters, blood lipid parameters, and the content of minerals in the blood and metacarpal bones.

## Material and methods

### Animals and experimental design

An experiment was carried out on 150 piglets at the age of 28 days of age (the day they were weaned), divided into five groups with similar body weight and sex. Each group comprised 30 piglets (15 gilts and 15 barrows), which were placed in 5 pens with 6 piglets in each (3 gilts and 3 barrows).

The study was conducted according to the guidelines of the Declaration of Helsinki. The experimental procedure was approved by the Local Ethics Commitee on Animal Experimentation of University of Life Sciences in Lublin, Poland (approval no. 21/2016). The piglets in each of the five groups were housed in a building with controlled environmental conditions and received crumble diet in an identical feeding system to about 30 kg body weight.

The control piglets (C) received a standard starter diet for piglets, as recommended by the NRC [13]. Piglets in group FR received a diet in which a portion of the soybean meal was replaced with an 8% share of dried fermented rapeseed meal (FRSM). Animals in group FR/FS received a diet in which a portion of the soybean meal was replaced with a 6% FRSM and a 2% share of dried fermented soybean meal (FSBM). Animals in group FS/FR received a diet in which a portion of the soybean meal was replaced with 6% FSBM and 2% FRSM. The last group of animals (FS) received a diet in which a portion of the soybean meal was replaced with 8% FSBM. FRSM and FSBM were obtained from European Protein AS (Bække, Denmark).

### Experimental diets

The ingredient composition of the experimental piglets’ diet is presented in Table 1.

**Table 1:** Ingredient composition (% of air-dry matter) of experimental diets [14].

Diet	Prestarter (29–42 days)					Starter (43–77 days)				
	Feeding group*					Feeding group*				
	C	FR	FR/FS	FS/FR	FS	C	FR	FR/FS	FS/FR	FS
Wheat	35.5	35.5	35.5	35.5	35.5	40.0	40.0	40.0	40.0	40.0
Barley	28.0	26.0	27.0	29.0	30.0	28.0	26.0	27.0	29.0	30.0
Soybean meal, 44% CP	16.0	10.0	9.0	7.0	6.0	16.0	10.0	9.0	7.0	6.0
Dried whey, 16% CP	4.0	4.0	4.0	4.0	4.0	0.0	0.0	0.0	0.0	0.0
Soybean oil	2.5	2.5	2.5	2.5	2.5	2.5	2.5	2.5	2.5	2.5
Complementary feed^1^^-^^5^	8.5	8.5	8.5	8.5	8.5	8.0	8.0	8.0	8.0	8.0
Mineral-vitamin premix^6^	5.0	5.0	5.0	5.0	5.0	5.0	5.0	5.0	5.0	5.0
Acidifier^7^	0.5	0.5	0.5	0.5	0.5	0.5	0.5	0.5	0.5	0.5
FRSM	0.0	8.0	6.0	2.0	0.0	0.0	8.0	6.0	2.0	0.0
FSBM	0.0	0.0	2.0	6.0	8.0	0.0	0.0	2.0	6.0	8.0

FSBM–fermented dried soybean meal, FRSM—fermented dried rapeseed meal

*Feeding group, C—control; FR—group receiving a diet with 8% FRSM; FR/FS—group receiving a diet with 6% FRSM and 2% FSBM; FS/FR—group receiving a diet with 6% FSBM and 2% FRSM; FS—group receiving a diet with 8% FSBM

^1^Complementary feed, 1 kg (control group) containing crude protein 36.15%, lysine 2.50%, methionine 0.73%, crude fat 14.3%, crude fibre 2.85%, calcium 1.2%, phosphorus 0.85%, sodium 0.35%, BHT 280 mg

^2^Complementary feed, 1 kg (group FR) containing crude protein 36.65%, lysine 2.61%, methionine 0.75%, crude fat 14.6%, crude fibre 2.75%, calcium 1.15%, phosphorus 0.84%, sodium 0.33%, BHT 280 mg

^3^Complementary feed, 1 kg (group FR/FS) containing crude protein 36.60%, lysine 2.58%, methionine 0.76%, crude fat 14.5%, crude fibre 2.77%, calcium 1.17%, phosphorus 0.84%, sodium 0.33%, BHT 280 mg

^4^Complementary feed, 1 kg (group FS/FR) containing crude protein 36.25%, lysine 2.50%, methionine 0.75%, crude fat 14.3%, crude fibre 2.85%, calcium 1.2%, phosphorus 0.84%, sodium 0.35%, BHT 280 mg

^5^Complementary feed, 1 kg (group FS) containing crude protein 36.05%, lysine 2.49%, methionine 0.74%, crude fat 14.3%, crude fibre 2.90%, calcium 1.22%, phosphorus 0.85%, sodium 0.36%, BHT 280 mg

^6^1 kg mineral-vitamin premix containing: calcium 130 g, phosphorus 50 g, sodium 35 g, magnesium 4.0 g, lysine 89 g, methionine 46 g, vitamin A 300,000 IU, vitamin D3 40,000 IU, vitamin E 2600 mg, calcium iodide 32 mg, selenium 8 mg, copper 3.2 g, iron 2.4 g, zinc 2.4 g, manganese 2976 mg, 25,000 FTU

^7^1 kg acidifier containing orthophosphoric acid 320 g, citric acid 110 g, fumaric acid 50 g, propionic acid 45 g, formic acid 45 g, carrier (silicon dioxide) 430 g.

The piglets were fed dry compound feed in accordance with NRC [13]. Piglets were provided *ad libitum* access to water. The nutrients and ANFs (Anti-nutritional factors) of the diets for the animals in the control and experimental groups are given in Table 2 [14].

**Table 2:** Analysed nutrients and bioactive substances in 1 kg of piglet diets (n = 3).

Diet	Prestarter (29–42 days)					Starter (43–77 days)				
	Feeding group*					Feeding group*				
	C	FR	FR/FS	FS/FR	FS	C	FR	FR/FS	FS/FR	FS
Dry matter, g	891.4	889.5	890.2	889.9	889.8	890.2	888.9	889.4	889.5	889.1
Crude ash, g	50.68	50.67	50.66	50.69	50.72	50.42	50.51	50.41	50.56	50.57
Crude protein, g	187.8	187.0	187.5	188.0	188.1	181.0	181.1	181.1	181.1	181.1
Ether extract, g	50.31	50.32	50.34	50.35	40.33	50.13	50.18	50.14	50.15	40.13
Crude fibre, g	40.07	40.05	40.05	40.03	40.02	40.41	40.28	40.22	39.98	39.95
Metabolizable energy^1^, MJ	13.32	13.32	13.31	13.32	13.32	13.21	13.21	13.21	13.21	13.21
Total phosphorus, g	6.81	6.82	6.82	6.81	6.81	6.78	6.75	6.78	6.76	6.77
Phytin phosphorus, g	3.92	2.44	2.73	2.82	2.74	3.93	2.46	2.76	2.87	2.79
Calcium, g	7.41	7.41	7.42	7.43	7.43	7.38	7.37	7.38	7.39	7.38
Glucosinolates, μmol g^-1^	0.081	0.330	0.174	0.103	0.062	0.081	0.331	0.175	0.104	0.063
Tannin, g kg^-1^	2.41	3.16	2.81	2.58	2.39	2.44	3.17	2.82	2.59	2.40
Lactic acid, g kg^-1^	14.21	81.54	80.16	75.83	74.94	14.01	81.34	79.93	75.15	74.65

FSBM–fermented dried soybean meal, FRSM—fermented dried rapeseed meal

*Feeding groups: C—control; FR- group receiving a diet with 8% FRSM; FR/FS—group receiving a diet with 6% FRSM and 2% FSBM; FS/FR—group receiving a diet with 6% FSBM and 2% FRSM; FS—group receiving a diet with 8% FSBM.

Metabolizable energy^1^ was calculated according to the equation of Kirchgessner and Roth [15].

### Laboratory analyses

#### Analysis of diets

Dry matter, crude ash, crude protein, crude fat, and crude fibre were determined in the diets [16]. Amino acids were analysed with a Sykam Amino Acid Analyzer (Laserchrom HPLC Laboratories Ltd. Inc., Rochester, UK) according to the AOAC procedure [16]. The content of calcium was determined by atomic absorption spectrometry with a Varian model 720-ES ICP-OES spectrophotometer (Varian, Palo Alto, USA), Total P content in the feed was determined colorimetrically with a Helios Alpha UV-Vis spectrophotometer (Spectronic Unicam, Leeds, UK). Also determined in the diets were the content of phytate phosphorus, lactic acid, glucosinolates and tannins [14].

Phytate was determined colorimetrically according Vaintraub and Lapteva [17]. The method of determining lactic acid was based on colorimetric determination of its content by the LA-Fe (III) complex method [18]. Glucosinolate content in sampls was estimated according to the standard PN-ISO 10633–1 [19] using high performance liquid chromatography. The Folin-Denis spectrophotometric method was used to determine the tannin content according to Canbaş et al. [20] in modification.

#### Blood and bone analysis

At 77 days of age, blood from 5 piglets from each group was collected for analysis. One barrow was taken from each pen for blood sampling and slaughter. Piglets from all pens had similar body weight/pen.

The animals had no access to feed for 12 hours before blood sampling. Blood was collected by a veterinarian from the jugular vein into heparinized 10 ml tubes.

The following parameters were determined in whole blood in an ABACUS-Vet analyser: haematocrit (Ht), haemoglobin content (Hb), red blood cell count (RBC), white blood cell count (WBC), and WBC differential, i.e. the percentages of granulocytes (GRA), lymphocytes (LYM), and the sum of eosinophils + basophils + monocytes (MID). The numerical ratio of granulocytes to lymphocytes (GRA/LYM) was calculated as well.

Cormay monotests were used for spectrophotometric determination of selected lipid parameters in the blood plasma, i.e. total cholesterol (CHOL), triacylglycerols (TG), HDL cholesterol, and total iron-binding capacity (TIBC). Low-density lipoprotein cholesterol (LDL) was calculated according to Friedewald’s et al. [21] formula.

Plasma titres of IgA, IgG, IgM and interleukin 6 (IL6) were determined using ELISA kits for porcine IgA, IgG and IgM (Bethyl Laboratories, Inc., Montgomery, TX) and for IL6 (Elabscience Biotechnology Co., Ltd).

At the end of the experiment, 5 piglets from each group (the same animals from which blood was collected) were sacrificed by electrical stunning inducing unconsciousness and exsanguination. The metacarpal bone was taken for analysis of mineral content. The right front foot was cleaned of all skin, muscle and connective tissue, and the third metacarpal bone was removed. Any remaining flesh adhering to the metacarpal was removed, and the bone was labelled and stored at −80°C until analysis. The third metacarpal bone was analysed for DM, ash, Ca, P, Cu, Zn and Fe content. The bone was placed in an oven at 100°C for 16 h to determine the dry matter weight. The sample was then ashed at 650°C in a muffle furnace, and the ash was digested in aqua regia (HCl/HNO_3_ mixture) and analysed for Ca and P. Phosphorus content was determined by spectrometry at 400 nm using a Helios Alpha UV-VIS spectrophotometer (Spectronic Unicam, Leeds, UK), according to AOAC [16].

Elements (Ca, Fe, Zn and Cu) were measured using an Analytik Jena PlasmaQuant PQ 9000 inductively coupled plasma optical emission spectrometer. The operational conditions, analytical lines, and wavelengths of the elements were as follows: RF generator power– 1200 W, RF generator frequency– 40.68 MHz, coolant gas flow rate– 12 L min^-1^, carrier gas flow rate– 0.5 L min^-1^, auxiliary gas flow rate– 0.6 L min^-1^, max, integration time– 15 s, pump rate–sample injection 19 rpm at normal mode (1 mL min^-1^) and flush fluid injection 78 rpm at fast mode (4 mL min^-1^), viewing configuration–Axial, replicates– 3, flush time– 30 s, wavelengths of absorption (resonance) lines [nm]– 422.7 for calcium, 238.2 for iron, 206.2 for zinc and 327.4 for copper.

### Statistical analysis

The numerical data were subjected to one-way ANOVA, and the significance of differences between groups was determined by the Tukey post-hoc test, assuming significance levels of 0.05 and 0.01. The tables present the means and the cumulative standard error of the mean (SEM). The calculations were made in SAS ver. 9.4 software (SAS Institute, Cary, NC).

## Results

The haemoglobin content in the blood of piglets from group FR/FS was significantly higher than in the control group (p ≤ 0.05). There were no significant differences between groups in the erythrocytes counts (RBC) and % Ht (p ≤ 0.05), (Table 3).

**Table 3:** Haematological parameters and immune parameters in piglet blood.

Item	Feeding group*					SEM
	C	FR	FR/FS	FS/FR	FS	
**Ht; %**	39.91	38.47	40.75	39.59	39.49	0.059
**Hb; mmol l**^**-1**^	5.53^b^	5.99^a^^b^	6.13^a^	5.92^a^^b^	5.90^a^^b^	0.668
**RBC; 10**^**12**^** l**^**-1**^	6.76	7.12	7.23	6.94	7.11	0.257
**WBC; 10**^**9**^ **l**^**-1**^	25.56^a^	22.55^b^	19.90^b^	25.59^a^	25.18^a^	0.448
**Leukogram**						
**LIM; %**	70.78	72.32	72.92	67.47	68.07	1.165
**MID; %**	1.08	1.03	0.900	0.717	0.850	0.052
**GRA; %**	28.13	26.65	26.18	31.82	31.08	1.163
**GRA/LIM**	0.407	0.373	0.369	0.478	0.476	0.024
**Immune parameters**						
**IgG; mg ml**^**-1**^	5.90^b^	6.35^a^	6.22^a^	5.99^a^^b^	6.06^a^^b^	0.109
**IgA; mg ml**^**-1**^	0.216^b^	0.299^a^	0.274^a^	0.246^a^^b^	0.240^a^^b^	0.012
**IgM; mg ml**^**-1**^	0.616^a^	0.551^a^^b^	0.496^b^	0.493^b^	0.482^b^	0.017
**Il6; pg ml**^**-1**^	164.4^a^	101.5^b^	94.2^b^	126.9^a^^b^	123.9^a^^b^	6.29

a, b—values in rows marked with different letters differ significantly at p ≤ 0.05.

*Feeding groups: C—control; FR—group receiving a diet with 8% FRSM; FR/FS—group receiving a diet with 6% FRSM and 2% FSBM; FS/FR—group receiving a diet with 6% FSBM and 2% FRSM; FS- group receiving a diet with 8% FSBM

RBC—red blood cells; Hb—hemoglobin content; Ht—haematocrit value; WBC—white blood cell count; GRA—granulocytes; MID—sum of eosinocytes, basocytes and monocytes; LIM–lymphocytes

Data are least squares means of 5 replicate pens per treatment.

Piglets receiving feed with a higher proportion of fermented soybean meal (groups FS/FR and FS) and from the control group (C) had significantly higher leukocyte counts (WBC) than the piglets in groups FR and FR/FS (p ≤ 0.05). The addition of a fermented rapeseed meal component to the feed significantly increased IgG and IgA titres compared to the control group (C), (p ≤ 0.05). The reverse pattern was noted for IL6 (C > FR = FR/FS). The IgM titre was higher in the control group than in the groups receiving FSBM (FR/FS, FS/FR and FS), (p ≤ 0.05), (Table 3).

Significantly higher total cholesterol (CHOL) content was found in the plasma of piglets from groups C and FR (8% share of FRSM) compared to groups FS/FR and FS (p ≤ 0.05). On the other hand, animals from groups C, FR and FS/FR had a significantly lower concentration of HDL cholesterol than animals from groups FR/FS and FS, and its percentage share of total cholesterol was lower as well (p ≤ 0.05). TG content in the plasma of piglets from group C, FR and FS/FR was significantly higher than in groups FR/FS and FS vs groups FR/FS and FS (p ≤ 0.05). The animals in the control group had a significantly higher concentration of LDL cholesterol compared to groups FS/FR and FS (p ≤ 0.05), (Table 4).

**Table 4:** Plasma lipid parameters in piglets.

Item	Feeding group*					SEM
	C	FR	FR/FS	FS/FR	FS	
**CHOL; mmol l**^**-1**^	2.14^a^	2.16^a^	2.07^a^^b^	1.88^b^	1.98^b^	0.032
**HDL; mmol l**^**-1**^	0.734^b^	0.834^b^	1.02^a^	0.777^b^	1.03^a^	0.026
**LDL; mmol l**^**-1**^	0.989^a^	0.904^a^^b^	0.787^a^^b^	0.746^b^	0.718^b^	0.032
**% HDL**	34.32^c^	38.66^c^	49.61^a^	41.78^b^	51.73^a^	1.44
**TG; mmol l**^**-1**^	0.919^a^	0.931^a^	0.582^b^	0.784^a^	0.521^b^	0.034

a, b, c—values in rows marked with different letters differ signify

cantly at p ≤ 0.05

*Feeding groups: C—control; FR—group receiving a diet with 8% FRSM; FR/FS—group receiving a diet with 6% FRSM and 2% FSBM; FS/FR—group receiving a diet with 6% FSBM and 2% FRSM; FS—group receiving a diet with 8% FSBM

CHOL—total cholesterol; HDL—high density lipoprotein cholesterol; LDL—low density lipoprotein cholesterol; TG–triacylglycerols

Data are least squares means of 5 replicate pens per treatment.

Piglets fed a diet with a fermented component based on rapeseed meal (groups FR, FR/FS and FS/FR) had significantly higher plasma concentrations of phosphorus and calcium compared to the control group (C), (p ≤ 0.05). Significantly higher levels of copper were found in the blood of pigs from group FS than in the control group (p ≤ 0.05).

Animals from groups FR, FR/FS and FS had significantly higher levels of zinc compared to the control group and group FS/FR (p ≤ 0.05). Plasma iron concentrations were significantly higher in piglets from groups FR and FR/FS compared to the control group (p ≤ 0.05). Significantly higher levels of TIBC were found in the plasma of pigs from groups FR, FR/FS and FS than in the control group (p ≤ 0.05), (Table 5).

**Table 5:** Mineral content in the blood plasma and metacarpal bone of piglets.

Item	Feeding groups					SEM
	C	FR	FR/FS	FS/FR	FS	
**Blood plasma**						
**Phosphorus; mmol l**^**-1**^	2.54^b^	3.21^a^	3.07^a^	3.12^a^	2.84^a^^b^	0.057
**Calcium; mmol l**^**-1**^	2.35^b^	2.50^a^	2.61^a^	2.50^a^	2.42^a^^b^	0.027
**Copper; μmol l**^**-1**^	19.29^b^	20.40^a^^b^	20.18^a^^b^	20.49^a^^b^	23.77^a^	0.480
**Zinc; μmol l**^**-1**^	9.38^b^	10.64^a^	11.73^a^	9.98^b^	11.30^a^	0.276
**Iron; μmol l**^**-1**^	34.60^b^	47.12^a^	47.57^a^	41.51^a^^b^	45.57^a^^b^	1.45
**TIBC; μmol l**^**-1**^	19.33^b^	22.76^a^	23.01^a^	20.98^a^^b^	22.87^a^	0.234
**Metacarpal bone (in dry matter)**						
**Phosphorus; g/kg**	69.34^b^	77.38^a^	76.45^a^	74.64^a^	75.98^a^	2.97
**Calcium; g/kg**	174.4^b^	186.5^a^	190.4^a^	190.1^a^	179.5^b^	4.53
**Copper; mg/kg**	7.92^b^	8.28	8.83	7.97	8.11	0.156
**Zinc; mg/kg**	119.87^b^	130.8^a^	133.8^a^	125.2^a^^b^	122.1^a^^b^	4.19
**Iron; mg/kg**	74.65^b^	76.76^a^	77.08^a^	75.67^a^^b^	74.65^b^	3.12

a, b—values in rows marked with different letters differ significantly at p ≤ 0.05

*Feeding groups: C—control; FR—group receiving a diet with 8% FRSM; FR/FS—group receiving a diet with 6% FRSM and 2% FSBM; FS/FR—group receiving a diet with 6% FSBM and 2% FRSM; FS—group receiving a diet with 8% FSBM; TIBC—total iron binding capacity

Data are least squares means of 5 replicate pens per treatment.

The inclusion of fermented feed in the diet (groups FR, FR/FS, FS/FR, and FS) significantly increased the phosphorus content in the metacarpal bone compared to the control group. The metacarpal bone of piglets from groups FR, FR/FS, and FS/FR had significantly higher levels of calcium and iron compared to the control group and group FS. Piglets receiving feed with a higher proportion of fermented rapeseed meal (groups FR and FR/FS) had significantly higher zinc content than the piglets from other groups (Table 5).

## Discussion

The inclusion of fermented components (8% FRSM or of FRSM in the amount of 6% and FSBM in the amount of 2%) in the diet of piglets significantly increased plasma immunoglobulin titres (IgG and IgA), which according to many authors may indicate stimulation of the humoral mechanism of active specific immunity [22]. Higher immunoglobulin titres may be the result of a competent immune response, e.g. to infection, or, as in this case, to stimulation of immunoglobulin production in piglets receiving a diet with a fermented component. Initially, the poor microbiota of the digestive tract in newborn piglets can be enriched by microbial strains (for example *Lactobacillus* sp.) found in fermented components. Exogenous strains, including lactic acid bacteria, adhere to the intestinal mucosa and can easily form a layer serving as a protective barrier, which helps piglets to achieve stabilization of the bacterial microbiota [23]. Li et al. [24] show that *L*. *fermentum* strains with properties similar to those of typical lactic acid bacilli (e.g. *Lactobacillus acidophilus*) also have a high capacity for adhesion to Caco-2 cell lines and are highly competitive with *Salmonella* and *E*. *coli*. Furthermore, better availability of zinc and copper leads to faster regeneration of the intestinal epithelium and increases levels of brush-border enzymes of enterocytes, which can positively affect the intestinal immune response [25]. Increased plasma content of IgA and IgG may also be associated with an increase in bioactive peptides resulting from the fermentation of rapeseed meal [23]. The increased amount of low-molecular-weight peptides in fermented rapeseed meal may be due to the partial digestion of high-molecular-weight peptides in rapeseed meal by proteases secreted by microorganisms associated with fermentation, such as *A*. *niger*), [9]. According to Xu et al. [1], bioactive peptides found in in fermented rapeseed meal may improve immune system function. Wang et al. [26] report that the addition of 3 g of low-molecular-weight peptides per kilogram of standard piglet diet increases immunoglobulin levels. Fazhi et al. [10] made similar observations in an experiment on ducks. According to Feng et al. [27], the inclusion of fermented soybean meal in the feed also stimulates the immune system, as demonstrated by an increase in IgM and IgA in the serum of broilers. However, no such reaction was observed in our experiment in piglets fed fermented soybean meal in the amount of 8 or 6% (groups FS and FS/FR). This may be because fermentation can degrade large proteins to small peptides, but mainly in rapeseed meal [28]. Yuan et al. [29] also observed no reaction to fermented soybean meal in piglet feed. In contrast, Zhu et al. [30], using various proportions of fermented soybean meal (from 5% to 15%) in piglet feed, noted a marked increase in plasma titres of IgG, IgM, and IgA. According to the authors, the reduction in glycinin (81.89%) and β-conglycinin (70.67%) in fermented soybean meal was the direct trigger for this reaction. Analysis of the effect of these two fermented feedstuffs (FRSM and FSBM) in our experiment indicates that the treatment with 6% dried fermented rapeseed meal and 2% dried fermented soybean meal (group FR/FS) seems to be the most effective in stimulating immune processes.

The inclusion of fermented components has also clearly been shown to reduce the amount of phytate phosphorus in the diet of piglets [31]. Many studies indicate that this could be associated with the activation of endogenous phytases in plant material [32]. According to Reale et al. [33], however, it is more likely that it creates more favourable pH conditions (lactic acid bacteria producing organic acids) for the activity of endogenous phytase. In addition, the fermentation process may have contributed to the degradation of the cell walls of the feed components, thus allowing greater access to the nutrients stored in them, including minerals [34]. This hypothesis is supported by the significant increase in the plasma and bone concentration of minerals, i.e. P, Ca, Zn and Fe, in the piglets receiving feed with dried fermented rapeseed meal (groups FR, FR/FS, and FS/FR). Such observations are reported by Xu et al. [23], who used different levels of fermented rapeseed meal in feed for broilers and noted an increase in their plasma P and Ca content. This is also confirmed by Fadel and El-Batal [35] and by El-Batal and Karem [36], according to whom fermentation, specifically the phytase-synthesizing microorganisms involved in it, has a role in reducing phytic acid in rapeseed meal. Shi et al. [9] report a reduction as high as about 86%. The reduction in phytic acid due to fermentation is consistent not only with our research, but also with the findings of El-Batal & Karem [36], who found that the presence of microorganisms accompanying the fermentation process and synthesizing phytase, such as *A*. *niger*, is the most significant effect of the process. This results in increased availability of P and Ca in a diet that includes fermented rapeseed meal, as well as divalent minerals, proteins and lipids bound in phytate complexes [9, 37]. High bioavailability of P, Ca and Zn also results in greater bone maturity and improvement of its mechanical properties [38, 39]. Zn not only can substitute for calcium in hydroxyapatite crystals, but is also closely linked to bone metabolism, especially during the stages of rapid growth, as it plays a crucial role as a catalyst of many enzymes that affect bone development and formation [40, 41].

The increase in the availability of iron, the key bioelement involved in erythropoiesis, in animals receiving a diet with 6% fermented rapeseed meal and 2% fermented soybean meal (FR/FS) was positively correlated with the increase in haemoglobin content in the blood. Better availability of iron corresponding to improved haematological blood parameters in piglets receiving a diet with 3% fermented soybean meal is reported by Kim et al. [42] and Kim et al. [43]. The authors also indicate that a higher proportion of fermented soybean meal reduces the level of haematological parameters, especially in older piglets. Chah et al. [44] made similar observations in broilers.

The reduction in the plasma levels of total cholesterol and LDL-cholesterol in the piglets receiving a diet with fermented components (FS/FR and FS) may have been linked to a decrease in the pH of the gastrointestinal tract contents, but primarily with an increase in the number of microorganisms responsible for the synthesis of numerous enzymes. These include lipase, responsible for the digestibility of lipid components, and phytase, protease, β-amylase, or β-glucanase, which improve nutrient availability and absorption. Microbial activity may also have resulted in binding of cholesterol and a decrease in faecal enzyme activity [45]. In addition, the probiotic microbes found in fermented products, by producing their metabolites, inhibit cholesterol esterification reactions in the intestinal mucosa and thus reduce the level of LDL-cholesterol in the body by uncoupling and precipitating bile acids. They are also thought to increase cholesterol excretion in the faeces by inhibiting the formation of easily digested fat micelles. *Lactobacillus acidophilus* and *Bifidobacterium* play the primary role in these functions [45]. Research by Hu et al. [46] in broilers, in which total serum cholesterol content was lower in birds whose diet included fermented rapeseed meal, indicates stimulation of metabolism of lipid compounds, including synthesis of low-molecular-weight HDL cholesterol. They suggest that fermented feed may act similarly to probiotics to lower cholesterol levels. The blood plasma triacylglycerol (TG) content reflects lipid metabolism in the body, and the reduced plasma TG content in animals fed fermented rapeseed and/or soybean meal (groups FR/FS and FS) indicates that they can significantly improve the utilization of fat in the diet. Chah et al. [44] made similar observations, indicating that broilers fed a diet with fermented soybean meal utilize lipid components more efficiently than broilers fed non-fermented soybean meal.

## Conclusions

In conclusion, the fermentation process enables better utilization of rapeseed or soybean meal by pigs. The inclusion of 8% fermented rapeseed meal or of fermented rapeseed meal in the amount of 6% together with fermented soybean meal in the amount of 2% in the diet of piglets seems to be the most beneficial variant for the mineral balance in the blood and bone as well as for stimulation of the immune system and haemoglobin synthesis. The differences observed in blood parameters between groups are interesting and may contribute to new prophylactic and therapeutic solutions in rearing of piglets. Therefore, dried fermented rapeseed meal could partially replace protein components from GMO crops (soybean meal) used in diets for pigs.
